# Impact of Quantum Dot Surface on Complex Formation with Chlorin e_6_ and Photodynamic Therapy

**DOI:** 10.3390/nano9010009

**Published:** 2018-12-22

**Authors:** Artiom Skripka, Dominyka Dapkute, Jurga Valanciunaite, Vitalijus Karabanovas, Ricardas Rotomskis

**Affiliations:** 1 Biomedical Physics Laboratory, National Cancer Institute, P. Baublio st 3b, LT-08406, Vilnius, Lithuania; artiom.skripka@emt.inrs.ca (A.S.); dominyka.dapkute@nvi.lt (D.D.); Jurgaval7@gmail.com (J.V.); Vitalijus.Karabanovas@nvi.lt (V.K.); 2 Life Science Center, Vilnius University, Sauletekio ave. 7, LT-10223 Vilnius, Lithuania; 3 Department of Chemistry and Bioengineering, Vilnius Gediminas Technical University, Sauletekio ave. 11, LT-10221, Vilnius, Lithuania; 4 Biophotonics Group of Laser Research Center, Faculty of Physics, Vilnius University, Sauletekio ave. 9, LT-10222, Vilnius, Lithuania

**Keywords:** photodynamic therapy, quantum dots, chlorin e_6_, energy transfer

## Abstract

Nanomaterials have permeated various fields of scientific research, including that of biomedicine, as alternatives for disease diagnosis and therapy. Among different structures, quantum dots (QDs) have distinctive physico-chemical properties sought after in cancer research and eradication. Within the context of cancer therapy, QDs serve the role of transporters and energy donors to photodynamic therapy (PDT) drugs, extending the applicability and efficiency of classic PDT. In contrast to conventional PDT agents, QDs’ surface can be designed to promote cellular targeting and internalization, while their spectral properties enable better light harvesting and deep-tissue use. Here, we investigate the possibility of complex formation between different amphiphilic coating bearing QDs and photosensitizer chlorin e_6_ (Ce_6_). We show that complex formation dynamics are dependent on the type of coating—phospholipids or amphiphilic polymers—as well as on the surface charge of QDs. Förster’s resonant energy transfer occurred in every complex studied, confirming the possibility of indirect Ce_6_ excitation. Nonetheless, *in vitro* PDT activity was restricted only to negative charge bearing QD-Ce_6_ complexes, correlating with better accumulation in cancer cells. Overall, these findings help to better design such and similar complexes, as gained insights can be straightforwardly translated to other types of nanostructures—expanding the palette of possible therapeutic agents for cancer therapy.

## 1. Introduction

Semiconductor quantum dots (QDs) have unique size-dependent optical properties and are used in biological and medical research [1], including the advancement of photodynamic therapy (PDT) of cancer [2]. PDT is a treatment method that combines light and light sensitive drugs—photosensitizers (PS)—to produce reactive oxygen species, which are deleterious to biological objects. Being non-invasive and highly selective, PDT is considered as an effective method to battle various types of malignancies with minimal damage to healthy tissues. The idea of using QDs for PDT is based on their ability to serve as Förster resonance energy transfer (FRET) donors to classical PS [2]. As energy donors, QDs possess all the necessary properties, such as broad absorption spectrum, tunable photoluminescence (PL) band position, high PL quantum yield (QY), long lifetime, and, most importantly, a far superior extinction coefficient as compared to classical PDT agents [3]. Combining these properties in a single donor, QD potentiates PS excitation and subsequently enhances the singlet oxygen generation efficiency [4,5,6,7,8]. Moreover, excitation of PS is restricted to use of visible light (typically in the red spectral side), hence to superficial applicability of PDT in tissues. Switching to near-infrared excitation, which coincides with optical transparency of tissues as in the case of two-photon excitation, enables PDT at greater tissue depths. In this regard, QDs could be used for two-photon PDT [9,10,11], as they have a large two-photon absorption cross section and remain photostable even at high laser excitation powers [12,13], in turn overcoming the two-photon excitation limits imposed on a variety of common PS.

Different QD–PS systems reported to date were designed by either covalent or non-covalent binding of PS [4,7,14,15]. In the latter case, the stability and subsequently FRET efficiency of the system highly depend on the interaction nature between QDs and PS. 

We have previously shown that the second-generation PS, chlorin e_6_ (Ce_6_), and QDs bearing phospholipid coating self-assemble into a stable complex with high FRET efficiency [16,17]. The major driving force of QD-Ce_6_ complex formation is the hydrophobic interaction between the non-polar moieties of amphiphilic Ce_6_ and phospholipids. Such a non-covalent QD-Ce_6_ complex retained high FRET efficiency in living cells and produced a significant phototoxic effect [18]. Additionally, we showed that the type of phospholipids in QD coating plays a crucial role in the formation and long-term stability of QD-Ce_6_ complex [19]. 

The importance of such and similar complexes lies in the simplicity of its formation and nature of the interaction, which results in exceptionally efficient energy transfer between the QDs and the bound PS. Moreover, it is valuable to understand if the self-assembly of such nanoparticle (NP)-PS complexes [20,21] could be directly translated in terms of varying the amphiphilic coating of NPs, how it would influence the indirect excitation of the PS, colloidal stability, accumulation in cells, and eventually the PDT effect.

In this work, we explore the QD-Ce_6_ complex by using QDs with two types of amphiphilic coating, namely lipids and polymers with differently charged terminal groups, to compare formation, stability, and FRET efficiency of QD-Ce_6_ complex in each case. To the best of our knowledge this is the first report that investigates QD-Ce_6_ complex formation with amphiphilic polymer coated QDs. Furthermore, we show the surface coating dependent accumulation of these complexes within breast cancer cells and its influence on PDT efficiency; this enables the current paper to go beyond photophysical studies and by direct comparison select an optimal QD-Ce_6_ formulation for the future of PDT.

## 2. Materials and Methods 

### 2.1. Materials

CdSe/ZnS QDs encapsulated with polyethylene glycol (PEG) grafted phospholipids (*L*-QDs), bearing amine or carboxyl functional groups, were purchased from eBioscience Inc. (Waltham, MA, USA). Amphiphilic polymer coated CdSe/ZnS QDs (*P*-QDs) with either PEG-amine or carboxyl group were purchased from Invitrogen Corp. (Carlsbad, CA, USA). All QDs had a photoluminescence (PL) band at around 605 nm. Chlorin e_6_ (Ce_6_) tetrasulfonic acid was obtained from Frontier Scientific Inc. (Logan, UT, USA). All materials were used without further purification. Stock solution of Ce_6_ was prepared by dissolving the powder in a small amount of 0.2 M NaOH solution and further diluting with phosphate buffer (PB) (pH 7). Working concentration of *L*- and *P*-QDs PB sols was 0.05 µM. QD:Ce_6_ molar concentration ratio was varied from 1:0.05 to 1:10, adding a small amount (5 μL) of Ce_6_ solution into 2000 μL of working QDs sol.

### 2.2. Spectral Studies

Steady-state absorption spectra were recorded with a Cary 50 UV-VIS spectrophotometer (Varian Inc., Palo Alto, CA, USA). PL measurements were carried out with a Cary Eclipse spectrophotometer (Varian Inc., Palo Alto, CA, USA). Fluorescence decay measurements were performed with a F920 spectrophotometer (Edinburgh Instruments, Livingston, UK), equipped with a single photon photomultiplier detector (S900-R) (Hamamatsu, Shizuoka, Japan) and a picosecond pulsed diode laser (EPL-405) (excitation wavelength 405 nm, pulse width 66.9 ps, repetition rate 2 MHz) (Edinburgh Instruments, Livingston, UK). Quartz cuvettes with the optical path length of 1 cm were used for all measurements.

### 2.3. Cell Culturing and Labeling

For intracellular localization of QDs, QD-Ce_6_ complexes and Ce_6_, MDA-MB-231 breast cancer cells (ATCC, USA) were seeded on a Nunc LabTek II chamber slide (Thermo Fisher Scientific, Waltham, MA, USA) at a density of 25,000 cells per well using standard cell culture medium (Dulbecco’s modified Eagle’s medium (DMEM), 10% fetal bovine serum (FBS), 1% penicillin/streptomycin antibiotic mix (all from Gibco, Waltham, MA, USA). Upon cell attachment, cells were treated with serum-free cell medium containing 10 nM of either different *P*-QDs or *L*-QDs or their QD-Ce_6_ complexes for 24 hours. In addition, 1 μM Ce_6_ solution was used as a control. Samples for confocal microscopy were prepared as previously described [22]. Briefly, cells were fixed with 4% paraformaldehyde solution (Sigma Aldrich, USA) and permeabilized with 0.2% Triton-X 100 (Sigma Aldrich, St. Louis, MO, USA). Actin filaments were stained with 15 U/mL Alexa Fluor 488 Phalloidin (Thermo Fisher Scientific, Waltham, MA, USA) and 25 μg/mL Hoechst 33342 (Sigma Aldrich, St. Louis, MO, USA) was used to label nuclei. 

### 2.4. PDT Studies 

To test the PDT effect, 10X concentrated QD-Ce_6_ complexes maintaining QD:Ce_6_ molar concentration ratio at 1:10 were prepared in a small amount of phosphate buffered saline (PBS) (pH 7.4) and kept at room temperature for 3 hours for complexes to equilibrate. Different QD-Ce_6_ sols in PBS were then diluted 10 times in a serum-free cell growth medium and poured over the cells grown on Nunc Lab-Tek chambered coverglass (Thermo Fisher Scientific, USA) at a density of 25,000 cells per well. After 24 hours the cells were irradiated with 470 nm light (MAX-302 xenon light source (Asahi Spectra, Japan)) providing a 17.7 J/cm^2^ irradiation dose in each well. After irradiation, cells were returned to the incubator. Twenty-four hours post treatment, 2 µM calcein AM and 4 µM ethidium homodimer-1 (LIVE/DEAD viability/cytotoxicity test kit by Thermo Fisher Scientific, Waltham, MA, USA) were added to each well and kept for 20 min before confocal microscopy analysis. Statistical analysis of PDT-affected cells was performed by direct counting of green (alive) and red (dead) cells in at least 5 different fields. Statistical significance was assessed using unpaired two-tailed Student’s *t* test.

### 2.5. Cellular Microscopy

Cell samples were imaged with a laser scanning confocal microscope (Nikon Eclipse TE2000-S, C1 Plus; Nikon, Japan) using either oil-immersion 60× NA 1.4 objective (Plan Apo VC; Nikon, Japan) for intracellular localization studies or dry 20× NA 0.5 objective (Plan Fluor; Nikon, Japan) for visualizing live/dead cells after the PDT. Diode laser (404 nm) excitation was used for Hoechst 33342, argon ion laser (488 nm) for Alexa Fluor 488 Phalloidin, calcein AM, and ethidium homodimer-1, and helium-neon laser (543 nm) for QDs. Images were captured with the EZ-C1 v3.90 image analysis software (Nikon, Japan) and processed using EZ-C1 Bronze v3.80 (Nikon, Japan) and ImageJ 1.48 (National Institute of Health, Bethesda, MD, USA) software. 

## 3. Results and Discussion

### 3.1. QD-Ce_6_ Complex Formation

In order to determine the effect of the QD coating on the complex formation with photosensitizer Ce_6_, we examined four different types of QDs. Namely, QDs with phospholipid (*L*-QDs) or amphiphilic polymer (*P*-QDs) coatings, bearing either amine or carboxyl terminal groups. It should be noted that all QDs except *P*-QD(carboxyl) were also grafted with PEG. 

**Figure 1:**
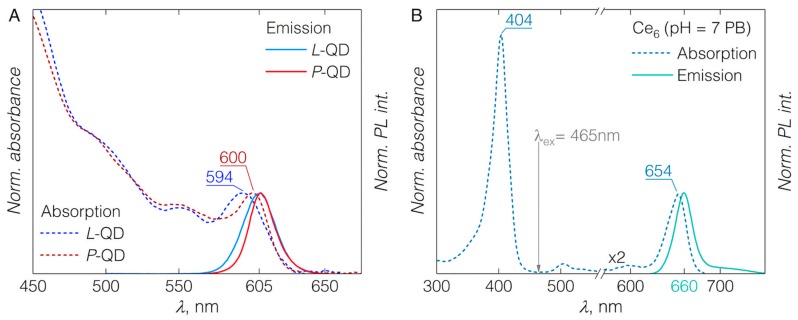
(**A**) Normalized absorption and emission spectra of quantum dots (QDs) functionalized with either phospholipids (*L*-QD) or amphiphilic polymer (*P*-QD) and bearing amine surface charge. (**B**) Normalized absorption and emission spectra of pure chlorin e_6_ (Ce_6_) in phosphate buffer (PB) (pH 7); note, the absorption region of Q(I) band is magnified for clarity; labels indicate absorption maxima position in nanometers. Emission spectra were measured with 400 nm excitation light. Arrow indicates excitation wavelength of 465 nm, used for Förster resonance energy transfer (FRET) studies.

The absorbance of QDs covered the major part of the visible spectrum starting from UV and terminating with the first excitonic band at 594 or 600 nm for *L*-QDs and *P*-QDs, respectively (Figure 1A). The PL band was centered at 605 nm for every QD studied. The PL band’s full width at half maximum was around 26 and 21 nm for *L*-QDs and *P*-QDs, respectively (Figure 1A). Absorption and PL properties of *L*-QDs or *P*-QDs were independent of the surface charge (Figure S1). Absorption spectrum of Ce_6_ solution showed the Soret band at around 404 nm and several less pronounced Q-bands, with the most intense Q(I) at 654 nm (Figure 1B). Emission of Ce_6_ in PB was centered at 660 nm.

**Figure 2:**
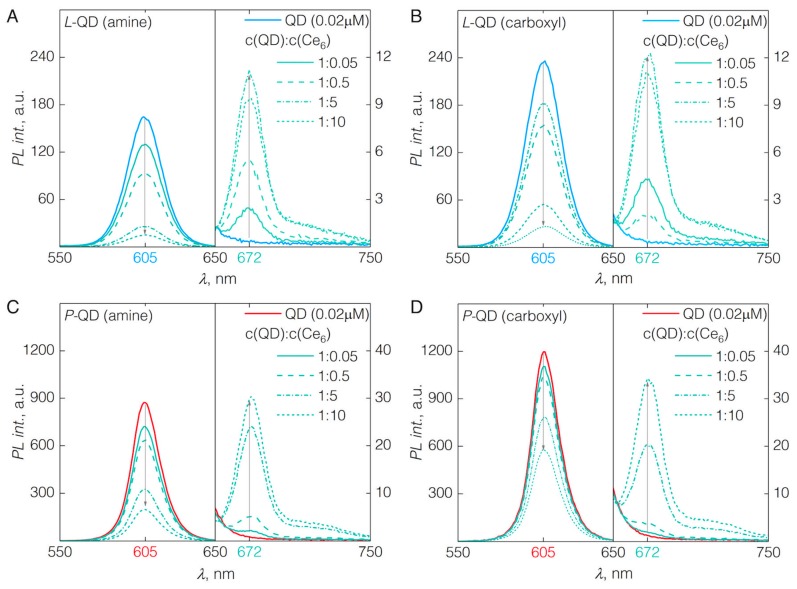
Emission spectra of pure QDs and respective QD-Ce_6_ complexes varying the molar concentration ratio between QDs and Ce_6_ from 1:0.05 to 1:10. Spectra were recorded under 465 nm excitation. (**A**) *L*-QD(amine)-Ce_6_; (**B**) *L*-QD(carboxyl)-Ce_6_; (**C**) *P*-QD(amine)-Ce_6_; (**D**) *P*-QD(carboxyl)-Ce_6_.

As shown previously [16,17], mixed together, QDs and Ce_6_ form a complex, resulting in spectral changes of both constituents. Most significantly, the fluorescence band of Ce_6_ shifts towards the red spectral side from 660 to 672 nm (Figure 2). Such a bathochromic shift is typically observed for Ce_6_ in non-polar milieu [23,24,25]. QDs’ coating—phospholipids or amphiphilic polymers—provide such non-polar microenvironment for bound Ce_6_ molecules. Furthermore, in the QD-Ce_6_ complex, the close proximity of Ce_6_ to the QD’s surface permits an efficient FRET [16]. The FRET from QDs to Ce_6_ in QD-Ce_6_ complex was confirmed by the fluorescence spectroscopy and fluorescence decay studies. When QD-Ce_6_ complex was excited at the wavelength (465 nm) solely absorbed by QDs (see Figure S2 for fluorescence excitation spectrum of Ce_6_), the emission bands of both, the QDs at 605 nm and the Ce_6_ at 672 nm, were observed—indicating FRET.

**Figure 3:**
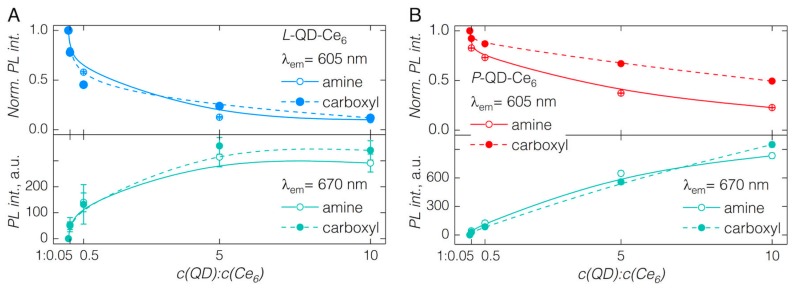
Normalized emission intensity of pure QDs and in the presence of increasing amount of Ce_6_ together with absolute fluorescence intensity of Ce_6_ after the binding to QDs. (**A**) *L*-QD-Ce_6_; (**B**) *P*-QD-Ce_6_. Line plots are introduced to guide the eye.

In both cases of *L*-QDs, irrespective of the surface charge, the formation of QD-Ce_6_ proceeded almost instantaneously after the addition of Ce_6_ (1 nM) to the QD sol (0.02 μM)—resulting in a quenching of QD PL band and appearance of Ce_6_ fluorescence at 672 nm (Figure 2A,B). Upon increasing the Ce_6_ concentration in the sol up to a QD:Ce_6_ molar concentration ratio of 1:10, the intensity of QD PL kept decreasing, and that of Ce_6_ reached a maximum at QD:Ce_6_ of 1:5 (Figure 2A,B; Figure 3A). Further increasing the amount of Ce_6_ in the sol resulted in the decrease of Ce_6_ fluorescence, probably due to the concentration quenching effect. Importantly, although carboxyl groups of Ce_6_ molecules are negatively charged, the QD-Ce_6_ complex formation happened in a similar fashion for both the positively charged *L*-QD(amine) and negatively charged *L*-QD(carboxyl). Hence, indicating that the hydrophobic interaction between the Ce_6_ and non-polar part of QD coating can overcome the potential barrier of the electrostatic repulsion.

Similarly, as in the case of *L*-QDs, the complex formation between Ce_6_ and QDs with the amphiphilic polymer coating (*P*-QDs) was observed by the bathochromic shift of the Ce_6­_ emission band to 672 nm and simultaneous quenching of the QD emission at 605 nm (Figure 2C,D). However, several differences in the *P*-QD-Ce_6_ complex formation were observed. In contrast to *L*-QD-Ce_6_, the quenching of QD PL (and subsequent increase of Ce_6_ PL) was considerably less pronounced when increasing the molar concentration of Ce_6_ in the sol, and the saturation effect of QD-Ce_6_ complex formation was not observed even at QD:Ce_6_ of 1:10. Differences of *P*-QD-Ce_6_ complex formation might stem from the possible crosslinking of the polymer coating [26,27], which would sterically hinder the intercalation of Ce_6_ within. Particularly in the case of *P*-QD(carboxyl), the interaction between the Ce_6_ and QDs was further impeded by the electrostatic repulsion, as the PL of carboxyl bearing QDs was significantly less quenched than that of their amine counterparts. Additionally, absence of PEG might have caused the slower complex formation dynamics with *P*-QD(carboxyl), however the exact influence of PEG in QD-Ce_6_ complex formation remains unknown and requires further investigation. 

It is worth noting that for the same crosslinking reasons, the amphiphilic polymer coating usually ensures better colloidal stability of NPs [26]. Thus, determined by the surface coating of QDs, a tradeoff exists between the dynamics of QD-Ce_6_ complex formation and its subsequent colloidal stability. We have additionally checked for the PL intensity changes during the first 16 min after the addition of the highest amount of Ce_6_ (QD:Ce_6_ = 1:10), which suggested that the QD-Ce_6_ complex is not obstructed from being formed in the case of *P*-QDs, rather it requires longer equilibration time (Figure S2, Figure 3). Notably, within the same time interval, PL intensity of *L*-QDs in QD-Ce_6_ complex decreased, alluding to a decreased colloidal stability of lipid coated QDs (Figure S2).

### 3.2. FRET from QDs to Ce_6_

After establishing the QD-Ce_6_ complex formation dynamics for differently coated QDs, we proceeded with estimation of FRET parameters for each QD:Ce_6_ molar ratio studied, at a time point when Ce_6_ is added to the sols. FRET efficiency was estimated from the QDs’ PL decay (Equation 1), accounting only for the dynamic quenching—non-radiative energy transfer.

(1) $$E=1-\frac{\langle \tau_{DA}\rangle }{<\tau_{D}>}$$

**Figure 4:**
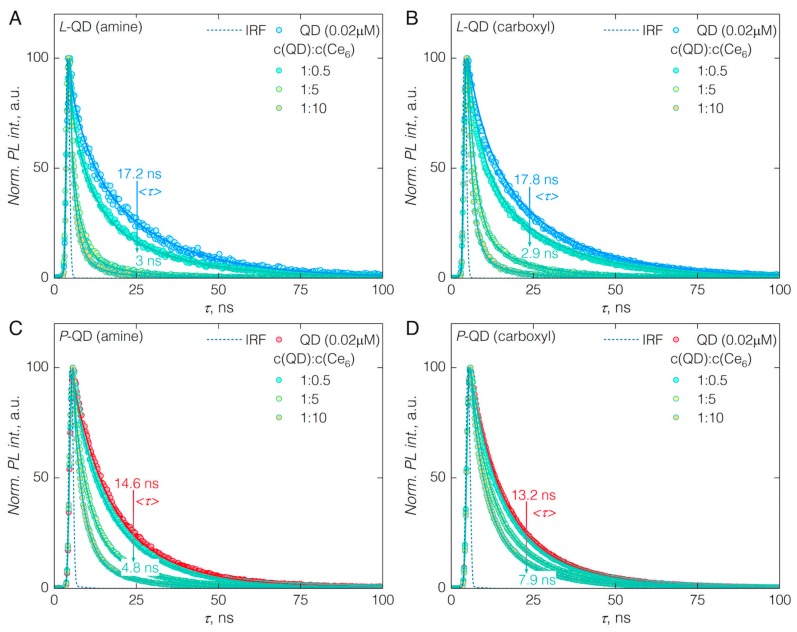
PL decay time (λ_ex_ = 405 nm; λ_em_ = 605 nm) of pure QDs and in the QD-Ce_6_ complex at QD:Ce_6_ molar concentration ratio of 1:0.5, 1:5 and 1:10. (**A**) *L*-QD(amine)-Ce_6_; (**B**) *L*-QD(carboxyl)-Ce_6_; (**C**) *P*-QD(amine)-Ce_6_; (**D**) *P*-QD(carboxyl)-Ce_6_. Amplitude-weighted lifetimes were obtained from tri-exponential fits of the PL decay curves. Instrument response function (IRF) is shown for each case.

Here, *<τ_D_>* and *<τ_DA_>* denote average photoluminescence decay time of donor (QDs) alone and in the presence of acceptor (Ce_6_), respectively. The average PL decay time for *L*-QDs decreased from 17.2 to 3 ns and 17.8 to 2.9 ns in the case *L*-QD(amine) and *L*-QD(carboxyl), respectively, at the highest concentration of Ce_6_ (Figure 4A,B). FRET efficiencies were around 82.8 and 83.7% for *L*-QD(amine)-Ce_6_ and *L*-QD(carboxyl)-Ce_6_, respectively (Table 1). High FRET values support the notion of Ce_6_ present in the phospholipid coating—in close proximity to the surface of QDs. In the case of *P*-QDs, addition of Ce_6_ to the *P*-QD(amine) sol, reduced the PL decay time from 14.6 to 4.8 ns, yielding 66.9% FRET efficiency, while for *P*-QD(carboxyl) it changed from 13.2 to 7.9 ns, hence 40.1% FRET efficiency (Figure 4C,D). Overall, FRET efficiencies obtained by exploiting hydrophobic interaction between Ce_6_ and amphiphilic coatings of QDs are highest reported thus far, when compared to other QD-Ce_6_ systems comprised of electrostatically or covalently interacting moieties [7,28].

In order to validate the presence of Ce_6_ close to the surface of QDs, we have determined the relative center-to-center distance *r* between different types of QDs and Ce_6_. PL *QY* for each type of QD was estimated *via* the comparative method, Rhodamine B served as a reference [29]; and obtained values (Table 1) were in line with those reported for QDs of similar structure [30,31]. The spectral overlap integral *J* and Förster distance *R_0_* between different QDs and Ce_6_ was calculated according to Equations 2 and 3, respectively [32].

(2) $$J=\frac{\sum F_{D}\left(\lambda \right)\varepsilon_{A}\left(\lambda \right)\lambda^{4}\Delta \lambda }{\sum F_{D}\left(\lambda \right)\Delta \lambda }$$

*F_D_* is the emission spectrum of QDs, *ε_A_* is the molar extinction coefficient of Ce_6_, and *λ* is the wavelength in nanometers.

(3) R0=0.211 (κ2n−4ΦDJ(λ))16

*Φ_D_* is the quantum yield of the QDs’ photoluminescence, *n* is the refractive index of the medium (here, 1.33), and *κ^2^* is a dipole orientation factor (here, 2/3—assuming random orientation of the QDs and Ce_6_ upon binding). The presence of multiple acceptors (Ce_6_) *m* with respect to a single donor (QD) was taken into account for *r* assessment (Equation 4).

(4) $$E=\frac{mR_{0}^{6}}{mR_{0}^{6}+r^{6}}$$

All of the obtained values are presented in Table 1. Relative *QY* was found to be similar between the same coating bearing QDs irrespective of the surface charge. Notably, *QY* of *P*-QDs was more than twice greater than that of *L*-QDs. While fairly similar PL band widths and peak positions of the investigated QDs yielded comparable spectral overlap values. 

Förster distance *R_0_* (representing the donor-acceptor separation at 50% FRET efficiency) on average was approximately 45 Å for *P*-QDs, as compared to 38 and 40 Å for *L*-QD(amine) and *L*-QD(carboxyl). Differences for higher *R_0_* values in the case of *P*-QDs can be directly attributed to higher QY of these QDs. Finally, the average donor-acceptor center-to-center separation *r* in QD-Ce_6_ complexes was around 42 and 45 Å for *L*-QD(amine)-Ce_6_ and *L*-QD(carboxyl)-Ce_6_, respectively. In the case of *P*-QDs, Ce_6_ was separated by 58 and 69 Å from *P*-QD(amine) and *P*-QD(carboxyl), respectively. It is worth noting that higher *r* values in the latter case represent a situation for which exact number *m* of bound Ce_6_ molecules is unknown due to slower binding kinetics, and thus are indicative only of the delayed complex formation (Figure S2). As a result, we have confirmed that complex formation is instantaneous between Ce_6_ and *L*-QDs, while it requires more time for Ce_6_ to intercalate into the amphiphilic polymer of *P*-QDs. 

When further deliberating about the presence of Ce_6_ in the coating of QDs, and subsequent FRET, it is worth noting that the refractive index specific to that microenvironment and not water has to be taken into account. The refractive index *n*, for phospholipids and amphiphilic polymers, might take values in the range of 1.4–1.6 [33,34]. We have checked for the effect of the refractive index on the *R_0_* and *r*, varying the value of *n* in Equation 3 from 1.33 to 1.6 (Figure S3A). With increasing *n*, *R_0_* decreased by 0.5 nm for the different QD-Ce_6_ complexes. Similarly, the *r* values decreased by 0.5 nm for both types of *L*-QD-Ce_6_ and 0.8 nm for *P*-QD-Ce_6_. Although these differences might appear small, it is important to recognize that Ce_6_ molecules can approach the surface of QD closer than thought initially. In fact, assuming the radius of *L*-QDs and Ce_6_ to be approximately 3.6 and 0.5 nm, respectively [35], the shortest possible distance between the two is within the calculated range of *r* = 3.8 ÷ 4.2 nm for *L*-QD-Ce_6_ (Figure S3C). On the other hand, although it is supposed that electric dipoles of QDs and bound Ce_6_ molecules have random orientation when energy transfer takes place, hence *κ^2^* is 2/3, we cannot completely rule out the possibility of preferential orientation of Ce_6_ when intercalated in the amphiphilic coating of QDs. Introducing this prospect (by varying *κ^2^* values in 2/3–4 range), we observe an increase in the *R_0_* and *r* values (Figure S3B), eluding to the possible greater separation between QDs and Ce_6_ to achieve the same energy transfer efficiencies, if specific—non-random—orientation could be assumed.

### 3.3. Cellular Accumulation of QD-Ce_6_ in Serum-Free Environment and PDT

To assess cellular internalization of QD-Ce_6_ complex and its PDT activity, we have performed *in vitro* testing of all four variants in the triple-negative basal-like breast cancer cell line MDA-MB-231, notorious for its aggressiveness and cancer stem-like properties—showing resistance to conventional cancer treatment methods [36,37]. 

**Figure 5:**
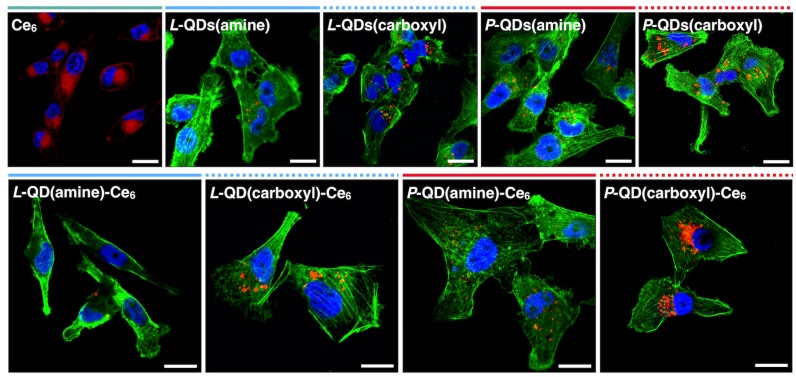
Accumulation of Ce_6_, QDs with various surface coatings, and their respective complexes in MDA-MB-231 breast cancer cells. Red—Ce_6_ (in the Ce_6_ image) or QDs (in all the QD and QD-Ce_6_ images); green—actin filaments; blue—nuclei. Scale bars in all images are 20 µm.

Notably, in the biological medium, surface charged QDs are rapidly coated with various proteins forming the so-called protein corona. Surface charge of QDs has an effect on the composition of the resulting protein corona, and their cellular uptake is often ascribed to the proteins assembled on the QDs’ surface [38,39]. To avoid serum-determined accumulation, QDs and QD-Ce_6_ complexes were incubated in the serum-free medium. QD-Ce_6_ complexes accumulated inside the cells (Figure 5) in the manner of pure QDs, rather than of Ce_6_. QDs are known to enter cells *via* endocytosis, localizing inside endocytic vesicles [40,41,42]. Meanwhile, Ce_6_ diffuses through the membrane and labels cells in a relatively uniform pattern with a slight selectivity to lipid membranes due to its amphiphilicity (Figure 5). We have observed that negatively charged QDs and their QD-Ce_6_ complexes were taken up by the cells noticeably better than their amine terminated counterparts. Additionally, cellular entry of QD-Ce_6_ complexes was influenced by the coating type, as *P*-QDs were internalized by cancer cells better than *L*-QDs. NPs’ surface chemistry greatly affects the efficiency of cellular accumulation and previous studies have showed that negatively charged NPs are internalized more rapidly than those of neutral or positive charge [43,44]. It is believed that carboxyl terminated QDs cluster at cationic sites of otherwise negative cell membrane, promoting NPs’ uptake. Additionally, suppressed accumulation of carboxyl terminated *L*-QDs might be explained by the presence of PEG. Due to its “stealth” properties, PEG reduces interaction with cellular membrane proteins, thus internalization of PEG coated NPs is restrained compared with non-PEGylated NPs [45]. 

**Figure 6:**
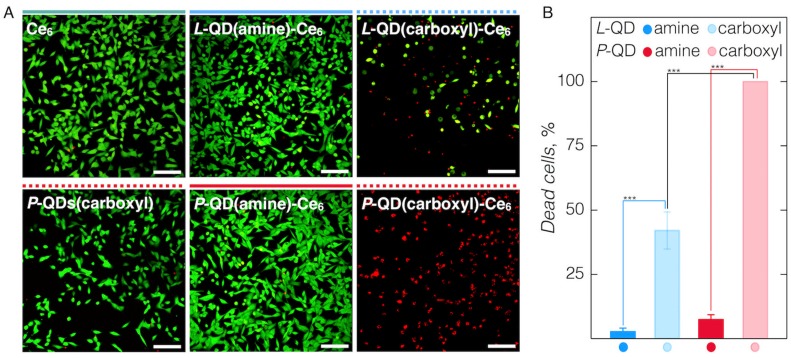
(**A**) Live (green)/Dead (red) images of cancer cells treated with different QD-Ce_6_ complexes for 24 h and subjected to 470 nm irradiation at 17.7 J/cm^2^ dosage. Images of the control experiments done with Ce_6_ or *P*-QD(carboxyl) alone are also presented. Scale bars in all images are 100 µm. (**B**) Respective percentages of dead cells in each studied case. Significant differences *p* < 0.0001 are indicated with asterisks.

We further tested the PDT effect of the QD-Ce_6_ complexes, induced solely *via* FRET—impinging light that is absorbed only by QDs and not Ce_6_. MDA-MB-231 cells, treated with various QD-Ce_6_ complexes, were irradiated under 470 nm light, and their viability was checked based on the number of alive (green) and dead (red) cells (Figure 6A). In correlation with cellular accumulation dynamics, PDT efficiency depended strongly on the type of the QDs’ coating. First, the PDT effect was evident only in cells treated with carboxyl-functionalized QD-Ce_6_ complexes. As cellular internalization of amine-functionalized QD-Ce_6_ complexes was restricted, these variants showed negligible phototoxicity towards cells. Second, *L*-QD(carboxyl)-Ce_6_ induced a 42% decrease in cancer cell viability and apoptotic-like morphology, while *P*-QD(carboxyl)-Ce_6_ demonstrated 100% phototoxicity (Figure 6B). As the formation of QD-Ce_6_ complexes was given enough time to equilibrate before being applied to cells, any differences in FRET activity could not account for the observed more efficient PDT, and thus were attributed solely to the better uptake of amphiphilic *P*-QD(carboxyl)-Ce_6_ inside the cells. It is also worth pointing out that despite the absence of covalent linking, Ce_6_ was retained within the coating of internalized QDs, since control cells treated only with *P*-QD(carboxyl) or Ce_6_, and irradiated with 470 nm light, remained unaffected and viable (Figure 6A).

## 4. Conclusions

We have explored the possibility of QD-Ce_6_ complex as a potential candidate for PDT, investigating the influence on the complex formation and activity of the QDs’ coating. Namely, negatively and positively charged QDs with phospholipid or amphiphilic polymer coatings were investigated. We have observed rapid QD-Ce_6_ complex formation for phospholipids bearing QDs, irrespective of their surface charge. While, QD-Ce_6_ complex with amphiphilic polymer coated QDs formed at a much slower rate. For each complex, effective energy transfer from QDs to Ce_6_ was observed. Following Förster’s formalism, we have determined that hydrophobic interaction brings Ce_6_ particularly close to the surface of QDs resulting in one of the most efficient FRET systems with an efficiency of up to 83.7%. *In vitro* experiments showed that only QD-Ce_6_ complexes made of carboxyl terminated QDs have significant PDT activity, in direct correlation with their enhanced cellular uptake.

Overall, we have decisively shown the versatility of non-covalent QD-Ce_6_ complexes, possible through a hydrophobic interaction of Ce_6_ with various amphiphilic coatings of QDs. These systems proved to be robust and possess high FRET efficiency, which can be exploited for indirect mediation of PDT and cancer eradication.

## Figures and Tables

**Table 1:** FRET parameters for different types of QD:Ce_6_ complexes at various Ce_6_ amounts (*m*).

Quantity	*L*-QD(amine)		*L*-QD(carboxyl)		*P*-QD(amine)		*P*-QD(carboxyl)	
*QY*	0.14		0.18		0.34		0.37	
*J*, 10^−13^ M^−1^cm^3^	1.16		1.19		1.26		1.22	
*R_0_*, Å	38.0		39.8		44.7		45.0	
*m*	*E*, %	*r*, Å	*E*, %	*r*, Å	*E*, %	*r*, Å	*E*, %	*r*, Å
0.5	23.9	41.1	20.8	44.3	10.9	56.5	5.4	64.7
5	74.9	41.5	70.6	45.0	47.3	59.5	24.4	71.1
10	82.8	43.0	83.7	44.5	66.9	58.3	40.1	70.7

## Supplementary Materials

The following are available online at http://www.mdpi.com/2079-4991/9/1/9/s1. Figure S1: Absorption and emission spectra of *L*- and *P*-QDs, Figure S2: Fluorescence excitation spectrum of Ce_6_, Figure S3: Change of *L*- and *P*-QD-Ce_6_ PL signals with time, Figure S4: *R_0_*, *r* values vs *n* and *κ^2^*, and schematic representation of QD-Ce_6_ complex.
