# Supplementary material for: Impact of Quantum Dot Surface on Complex Formation with Chlorin e6 and Photodynamic Therapy
Source: Nanomaterials (Basel). 2018 Dec 22;9(1):9. doi: 10.3390/nano9010009 (PMC6359007; doi:10.3390/nano9010009)
Supplement: Supplementary file 1 [file nanomaterials-09-00009-s001.pdf]

nanomaterials

Supplementary Materials

Impact of Quantum Dot Surface on Complex Formation with Chlorin e6 and Photodynamic Therapy

Artiom Skripka [1], Dominyka Dapkute [1,2], Jurga Valanciunaite [1], Vitalijus Karabanovas [1,3] and Ricardas Rotomskis [1,4*]

[1] Biomedical Physics Laboratory, National Cancer Institute, P. Baublio st 3b, LT-08406, Vilnius, Lithuania
[2] Life Science Center, Vilnius University, Sauletekio ave. 7, LT-10223 Vilnius, Lithuania; artiom.skripka@emt.inrs.ca (A.S.); dominyka.dapkute@nvi.lt (D.D.); Jurgaval7@gmail.com (J.V.); Vitalijus.Karabanovas@nvi.lt (V.K.)
[3] Department of Chemistry and Bioengineering, Vilnius Gediminas Technical University, Sauletekio ave. 11, LT-10221, Vilnius, Lithuania
[4] Biophotonics Group of Laser Research Center, Faculty of Physics, Vilnius University, Sauletekio ave. 9, LT-10222, Vilnius, Lithuania
* Correspondence: ricardas.rotomskis@nvi.lt; Tel.: +370-5-219-0908

Spectral properties of QDs

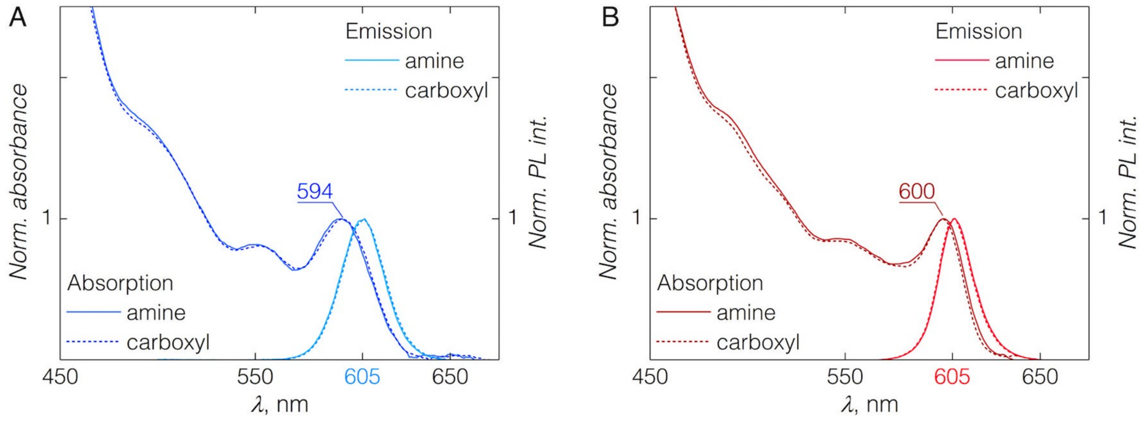

Figure S1. Normalized absorption and emission spectra of QDs functionalized with either phospholipids (*L*-QDs) (**A**) or amphiphilic polymer (*P*-QDs) (**B**) and bearing amine or carboxyl surface charge.

Fluorescence excitation of Ce_6

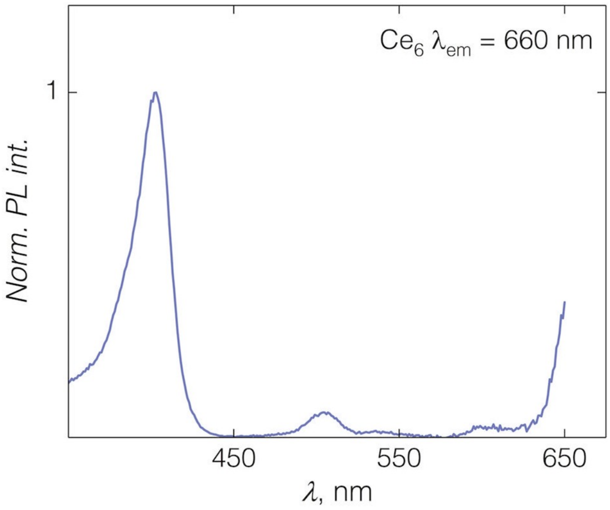

Figure S2. Normalized fluorescence excitation spectrum of Ce_6 in phosphate buffer (pH=7), measured at emission wavelength of 660 nm.

Complex equilibration dynamics

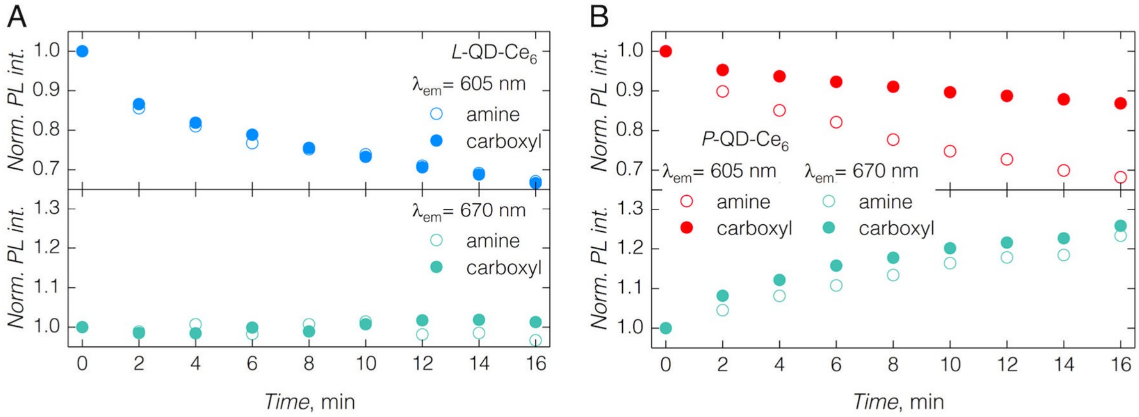

Figure S3. Temporal change of the QD and Ce_6 PL intensity in the QD-Ce_6 complex after its initial formation. (**A**) – Represents the normalized PL intensity changes in *L*-QD-Ce_6 complex composed out of amine/carboxyl bearing *L*-QDs; (**B**) – in case of amine/carboxyl bearing *P*-QDs.

Influence of n and κ^2 on R_0 and r

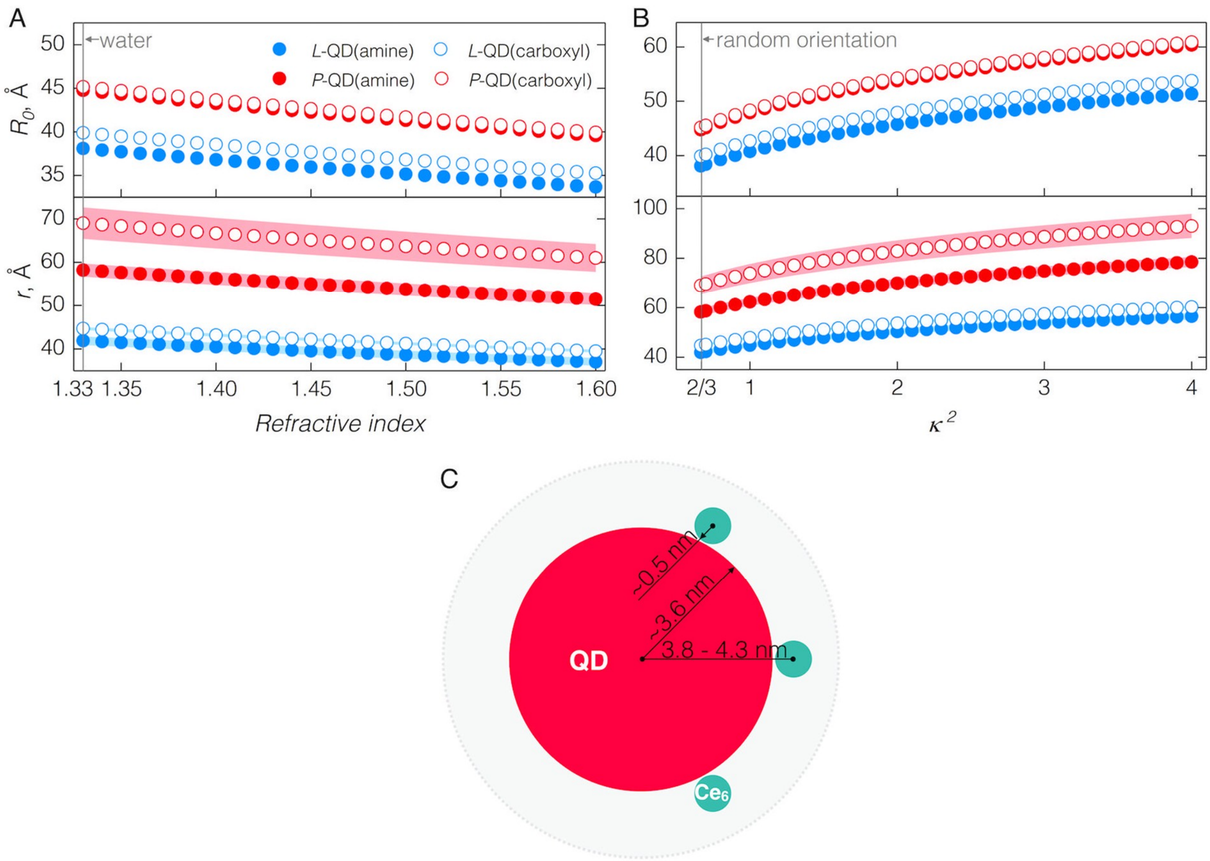

Figure S4. Change of the Förster distance R_0 (top) and the center-to-center distance between QDs and Ce_6 r (bottom) as a function of the refractive index of the medium (**A**; when κ^2 = 2/3) or orientation factor κ^2 (**B**; when n = 1.33). Center-to-center distances between the different QDs and Ce_6 were averaged taking values for different amounts (m) of Ce_6. Errors of r are represented by the shaded areas. (**C**) – schematic representation of the Ce_6 in the amphiphilic coating of QDs and the possible center-to-center separation between the two.
